# Efficacy and safety of cimicoxib in the control of perioperative pain in dogs

**DOI:** 10.1111/jsap.12082

**Published:** 2013-06

**Authors:** E Grandemange, S Fournel, F Woehrlé

**Affiliations:** Vétoquinol Research CentreBP189, 70204, Lure cedex, France

## Abstract

**Objectives:**

**To determine the efficacy and safety of cimicoxib (Cimalgex®; Vétoquinol SA) for the control of perioperative pain in dogs.**

**Methods:**

**A double-blind, randomized, controlled multi-centre field study was conducted in 237 dogs undergoing orthopaedic or soft tissue surgery. Pain was monitored by the attending veterinarian over the 7 days following the surgical procedure using two pain-scoring systems and a visual analogue scale. An enhanced monitoring protocol for postoperative pain was utilized during the first 24 hours after surgery. The dog owner's assessment of perceived analgesia during this time period was also recorded.**

**Results:**

**Cimicoxib demonstrated statistically significant non-inferiority compared to carprofen. These findings were confirmed by owners’ assessments and by the evolution of the pain scores. Both drugs were well tolerated throughout the study.**

**Clinical Significance:**

**Cimicoxib had non-inferior efficacy and tolerability when compared to carprofen for the control of perioperative pain in dogs undergoing orthopaedic or soft tissue surgery.**

## Introduction

The welfare implications associated with postoperative pain are self-evident but other complications such as delayed wound healing or an increased risk of self-trauma of the surgical site (which may result in chronic pain) cannot be ignored (Conzemius *et al*. 1997, Taddio *et al*. 1997). Non-steroidal anti-inflammatory drugs (NSAIDs) are an extremely valuable component of perioperative protocols by virtue of their duration of action, safety profile and efficacy as analgesics for both soft tissue and orthopaedic procedures. Benefits ascribed to preoperative administration are a potential pre-emptive effect and the presence of analgesia during the recovery phase (Lascelles *et al*. 2005, Tranquilli *et al*. 2007).

NSAIDs are currently the most widely used therapeutic class in veterinary medicine and a number of these drugs have been shown to be effective analgesics, including carprofen (Rimadyl®; Pfizer AH) (Grisneaux *et al*. 1999) and tolfenamic acid (Grandemange *et al*. 2007). NSAIDs are also anti-inflammatory and antipyretic.

NSAIDs exert non-specific effects by inhibiting isoforms of cyclooxygenase (COX) synthase which synthesize prostaglandins from arachidonic acid. At least two isoforms of COX exist: COX-1 and COX-2. The majority of acute and chronic toxicities associated with NSAIDs, such as gastrointestinal ulceration and disruption of platelet aggregation, are thought to be associated with COX-1 inhibition (Rivière *et al*. 2009). As COX-2 is the principal enzyme responsible for the overproduction of prostaglandins following acute injury or infection, it is postulated that drugs with greater specificity for the COX-2 isoform may be more effective analgesics but, to date, this theory remains unproven (Muir 2009). NSAID molecules with increased specificity for COX-2 are known as coxibs. Examples of this group are available in the veterinary markets of Europe and North America (e.g. firocoxib, deracoxib and robenacoxib). Their efficacy and safety have been demonstrated by field trials and experimental pain models (Pollmeier *et al*. 2006, Schmid *et al*. 2009).

Cimicoxib (Cimalgex®) is the newest member of the coxib family and was developed to assist in the management of pain and inflammation in companion animals. This study was designed to evaluate the perioperative analgesic efficacy and safety of cimicoxib in comparison to carprofen when administered to dogs before surgery and during the subsequent postoperative period.

## Materials And Methods

### Experimental design

A double-blind, randomized, parallel controlled, multi-centre study was carried out in 28 veterinary practices throughout France, Germany and Spain. Approval was granted by the appropriate regulatory authorities and informed consent was obtained from all pet owners. The study design conformed to local animal welfare standards and complied with the Guidelines for Good Clinical Practice (VICH 2000) and the European Medicine Agency (EMA) Guidelines for the Conduct of Efficacy Studies for NSAIDs (EMEA 2001a).

Induction of anaesthesia was defined as T0, and extubation (or the end of the surgical procedure if inhalational anaesthesia was not administered), was defined as T1. Dogs were evaluated for pain at least 2 hours before T0 and at subsequent time intervals of 1, 4, 12, 24, 72 and 168 hours after T1.

### Selection of animals

Dogs were eligible for inclusion if they were older than four months of age, unrestricted by weight or sex and scheduled to undergo either orthopaedic or soft tissue surgery where a painful postsurgery component was anticipated (Table 1). They had not received short-acting anti-inflammatories (such as NSAIDs) during the week before T0, or long-acting corticosteroids within a 2-month period preceding T0. Patients were excluded if they were pregnant or lactating or had evidence of coagulopathies or cardiac, renal or hepatic disease. A history of gastric ulceration also precluded enrollment.

**Table 1:** Pre-emptive scoring system to anticipate the amount of pain induced by surgical procedures (Mich & Hellyer 2009)

Minor procedures: no pain or temporary pain	Moderate surgeries: moderate pain
Grooming	Anal sacculectomy
Nail trim	Cutaneous mass removal
Physical examination, restraint	Cystotomy
Radiography	Dental extraction
Suture removal, cast application, bandage change*	Ovariohysterectomy, castration, caesarean section
	Severe laceration repair
Minor surgeries: minor pain	Major surgeries: severe pain
Abscess lancing	Ear canal ablation
Dental cleaning	Fracture repair, cruciate ligament repair
Ear examination and cleaning	Limb amputation
Removal of cutaneous foreign bodies	Thoracotomy, laminectomy
Suturing, debridement Urinary catheterization	Exploratory laparotomy

* Setting of fractures and some bandage changes can be very painful

### Clinical examination

**FIG 1:**
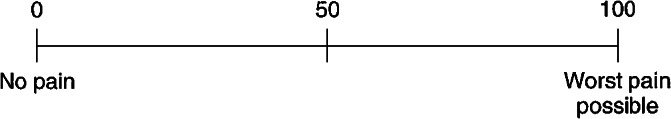
Visual analogue scale used to estimate an animal's current pain status

Pain was assessed by concomitant use of a visual analogue scale (VAS) and two separate pain-scoring systems at intervals from 2 hours before T0 through to 168 hours postsurgery. For a given 100 mm VAS line, 0 mm represented ‘absence of pain’ and 100 mm indicated ‘worst pain possible’ (Fig 1) (Mich & Hellyer 2009). The VAS assessment was combined with pain evaluation based upon observation of the dog both at rest and following stimulation of the painful area (when not contra-indicated). This provided an overall pain score based on observed clinical parameters (Table 2). An additional pain score was derived using the 4AVet system (Table 3) and sedation was simultaneously quantified as: [0] absence of sedation, [1] slight sedation, [2] moderate sedation, [3] marked sedation. On the final day of the study (168 hours post-T1), both the veterinarian and the dog's owner recorded their assessment of the quality of analgesia provided by the study drugs. Veterinarians were asked to score perceived analgesia as excellent, good, adequate, or inadequate while owners provided a subjective assessment of analgesia using the terms very satisfactory, satisfactory, not very satisfactory or not at all satisfactory.

**Table 2:** Clinical parameters

Parameter	Scoring system
Overall pain score: all time points	
Heart rate (/min)	Increase when compared with the preanaesthesic period
	[0] ≤10%
	[1] 11-30%
	[2] 31-50%
	[3] >50%
Behavioural response	[0] happy dog, plays, reacts with enthusiasm when called
	[1] alert dog, clearly responds when called
	[2] anxious dog, reduced response when called
	[3] abnormally restless dog, aggressive if approached or: abnormally depressed dog, showing no response
Pain on manipulation or pressure of the surgical site	[0] no reaction
	[1] tries to escape from manipulation
	[2] tries vigorously to escape, vocalizes
	[3] manipulation is unbearable, aggressive response
Vocalizes	[0] no vocalization
	[1] stops vocalizing when comforted by voice
	[2] persistent vocalization, despite com forting by voice
Other Clinical Parameters: All time points	
Respiratory rate (/min)	[0] normal
	[1] slight abdominal effort
	[2] marked abdominal effort
Movement	[0] normal to exuberant: active dog that moves with energy, able to jump
	[1] dog almost able to move normally
	[2] dog reluctant to move, adopting postures to relieve discomfort
	[3] dog barely moving, very reluctant to stir
Rectal temperature	in °C
Appetite and surgical site assessment: 24 hours post T1	
Appetite	[0] dog eating with enthusiasm on presentation of food
	[1] dog eating adequately
	[2] reduced appetite
	[3] dog anorexic
Oedema of the operative area	[0] absent
	[1] slight
	[2] moderate
	[3] severe
Inflammation of the operative area	[0] absent
	[1] slight
	[2] moderate
	[3] severe

**Table 3:** 4A-Vet scoring system

Subjective overall assessment	Absence of pain	0
		1
		2
	Intolerable pain	3
General behaviour	Among the following symptoms:	
	shows respiratory alterations	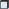
	vocalizing	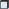
	crouched/stooped posture	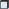
	unable to move	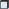
	restless and/or depressed	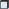
	loss of appetite	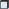
	looks at, chews/licks the surgical site	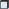
	lame, moves about with difficulty or is reluctant to move about	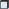
	- No sign present	0
	- Only 1 sign present	1
	- 2 to 4 signs present	2
	- 5 to 8 signs present	3
Interactive behaviour	Is attentive and responds to touch/voice	0
	Timid/nervous response	1
	Does not respond immediately	2
	Does not respond or responds aggressively	3
Heart rate	<10% increase	0
	11-30% increase	1
Initial value	31-50% increase	2
	>50% increase or cannot be assessed	3
Reaction at palpation or manipulation of surgical site	No visible or audible response	
	- after 4 tests	0
	Visible or audible response(s)	
	- at the fourth	1
	- at the second and third	2
	- at the first test	3
Intensity of this response	No response	0
	Responds easily, tries to escape	1
	Turns head or vocalizes	2
	Aggressive response or non-responsive	3
Total score	1 to 5 : slight pain	
	6 to 10 : moderate pain	
	11 to 18 : severe pain	

Each practice used a common anaesthetic protocol for all patients in order to eliminate disparities in analgesia associated with variations in methodology. Although premedication with sedatives with a significant analgesic component (e.g. medetomidine, xylazine) and analgesics (such as opioids) was not permitted, practitioners could withdraw the dogs from the study if they felt rescue analgesia was required (no cut off limit, rescue analgesia of their choice). All adverse events were reported, irrespective of a potential causative link to the NSAID used.

### Laboratory examination

Blood samples were collected before inclusion, at 24 and 168 hours following T1. Analysis by a local reference laboratory provided routine haematology biochemistry and coagulation profiles (Table 7).

### Treatment administration

Dogs were randomized to receive either 2 mg/kg cimicoxib (Cimalgex®, Vétoquinol SA) or 4 mg/kg carprofen treatment in a 1:1 ratio. The randomization was stratified based upon the type of surgical procedure and the practice. Cimicoxib was administered as oral tablets 2 hours before T0, and thereafter at 24 hour intervals for 2 days. Following the 72-hour clinical assessment, the treatment could be extended by 4 days if the veterinarian deemed it necessary for the control of residual postoperative pain. Carprofen (Rimadyl® for injection; Pfizer AH) was administered as a single preoperative injection 2 hours before T0, followed by five oral doses at 24-hour intervals as recommended in the European Marketing Authorization of Rimadyl® tablets (Rimadyl® F; Pfizer AH).

Treatment administration was blinded using the dual investigator method. One investigator (the clinician) performed clinical examinations and pain assessments and a second investigator (the drug dispenser) was responsible for product management and administration.

### Assessment criteria

The primary efficacy criterion was the observed postoperative pain, assessed by several different methods over two discrete time periods: the initial 24 hour period and 24 to 168 hours following T1. Observable pain for the initial 24 hour postoperative period was quantified by plotting the values obtained by the 4AVet scoring system (preoperative assessment to 24 hours after T1) and calculating the area under the curve (AUC) using a trapezoidal method. For the remainder of the study, observed postoperative pain was assessed by “success rate”, defined as the percentage of dogs for which analgesia after treatment was considered as “good” or “excellent” at 168 hours postsurgery. If cases required rescue analgesia they were automatically scored as “inadequate analgesia”.

Secondary endpoints, included to confirm the validity of the conclusions regarding treatment efficacy, were the time courses of VAS and overall scores of pain.

### Statistical analysis

As suggested by the EMA statistical principles (EMA 2001b), a non-inferiority approach was used to compare treatments for the primary efficacy criterion (for both time periods). The primary aim of a non-inferiority trial is to demonstrate that the response to the investigational product is not clinically inferior to a comparative agent. This is usually achieved by showing that the true treatment difference is likely to rest above a lower limit of clinically relevant differences (EMA 2001b). To account for the high variability of pain, a 20% margin was selected as being a clinically acceptable difference to test cimicoxib's non-inferiority to carprofen. As two primary endpoints depending on the time period were retained, a non-inferiority statistical analysis was performed for each endpoint. For the first time period (where the 4AVet pain score AUCs were compared), the difference between the treatment groups was calculated together with a two-sided 90% confidence interval after log transformation of the AUCs to normalize their distribution. If the upper bound of the confidence interval of the ratio of group means (cimicoxib mean/carprofen mean) was greater than 1·25, inferiority was not rejected. The analysis of the AUC mean ratios was conducted according to the EMA guidelines for bioequivalence (EMA 2001c). For the second time period (comparison of success rates), inferiority was rejected if the lower bound of the 95% confidence interval of the observed difference between success rates (cimicoxib success rate – carprofen success rate) was greater than −20%. The secondary endpoints were analysed by the mixed procedure with repeated measures of SAS (fixed effects: treatment, examination time, time × treatment interaction). Comparisons at baseline were done using chi-square tests or Kruskal-Wallis tests, depending on the characteristics of the analysed variable. All statistical calculations were performed with SAS software (SAS/STAT 9.1) with the individual dog forming the experimental unit. Before starting the study, an 80% power calculation indicated that 100 dogs per group were required based on the following hypotheses: one-sided test, first type error = 2·5%, non-inferiority margin = 20%, response rate (excellent + good analgesia) = 70% in both treatment groups.

## Efficacy Results

### Evaluation and inclusion of patients

A total of 248 animals were presented for inclusion; 11 were excluded because of biochemical abnormalities noted on preoperative screening. The remaining 237 dogs were enrolled across 28 veterinary practices and divided into cimicoxib (n=114) and carprofen (n=123) treatment groups. Dogs of both sexes were enrolled in the study and similar demographic characteristics (P≥0·12) were present in each treatment group (weight and age presented in Table 4). No differences in any clinical parameter were detected at inclusion (P≥0·24). A similar distribution of procedure type and surgical duration was observed for both treatment groups (Table 4: P≥0·47) with the operations conducted (n=119 soft tissue; n=118 orthopaedic) being representative of commonly conducted procedures in general practice. For the cimicoxib group, 34·2% dogs were considered to require the additional 4 days pain relief at their 72 hour postoperative assessment. Of these dogs, 61·5% had undergone orthopaedic surgery.

**Table 4:** Overview of surgical procedures and of some demographic data

	Cimicoxib	Carprofen	Test
*Age (months)*			Kruskal-Wallis test
Mean (Sd)	62·7 (47·4)	60·3 (47·2)	P=0·65
Median (range)	51 (5–180)	48 (5–186)	
*Weight (kg)*			Kruskal-Wallis test
Mean (sd)	21·2 (12·3)	22·9 (12·4)	P=0·22
Median (range)	19·1 (2·2–65·0)	22·3 (3·0–69·0)	
*Type of surgery – N=237*			Chi-square test
Orthopaedic (%)	54 (47·4)	64 (52·0)	P=0·47 n.s
Soft tissues (%)	60 (52·6)	59 (48·0)	
*Orthopaedic*			
Coxofemoral joint (%)	5 (9·3)	12 (18·8)	
Patella/cruciate ligaments (%)	29 (53·7)	25 (39·1)	
Fracture (%)	18 (33·3)	18 (28·1)	
Others (%)	2 (3·7)	9 (14·1)	
*Soft tissues*			
Castration/urogenital surgery (%)	39 (65·0)	36 (61·0)	
Soft tissue tumour excision (%)	6 (10·0)	12 (20·3)	
Mastectomy (%)	8 (13·3)	7 (11·9)	
Others (%)	7 (11·7)	4 (6·8)	
*Surgery duration*			
*Soft tissue (min)*			
Mean (sd)	79·8 (47·7)	70·6 (56·9)	
Median (range)	62·0 (19–225)	55·0 (15–320)	Pooled analysis
*Surgery duration*			Kruskal-Wallis test
*Orthopaedic (min)*			P=0·56 n.s
Mean (sd)	94·5 (47)	103·4 (53·6)	
Median (range)	86·0 (30–250)	92·5 (30–287)	

### Efficacy assessments

**FIG 2:**
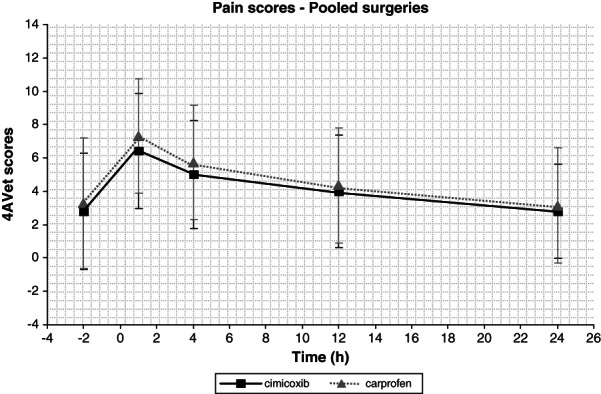
Time course of the 4A-Vet score from 2 hours before surgery until 24 hours postsurgery (pooled surgeries, mean and sd, rejection of inferiority P<0·0005)

**FIG 3:**
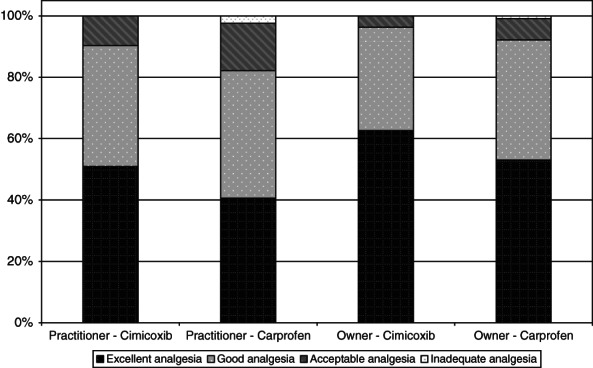
Assessment of the quality of analgesia at the end of the follow-up period by the clinical investigator (the practitioner) and the owner. There was no statistical difference between the two tested molecules (P=0·13 for the practitioner's feed back; P=0·32 for the owner's scores)

**FIG 4:**
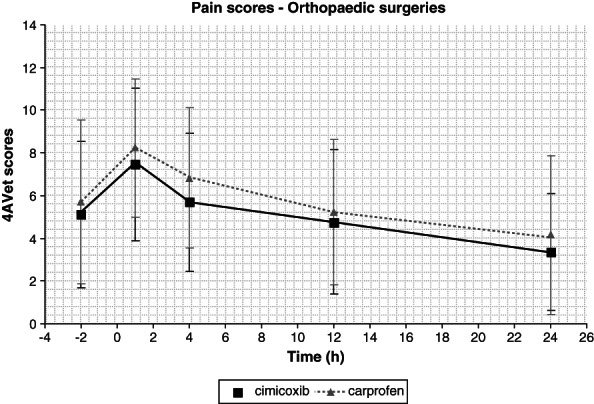
Time course of the 4A-Vet score from 2 hours before surgery until 24 hours postsurgery (orthopaedic surgeries, mean and sd)

**FIG 5:**
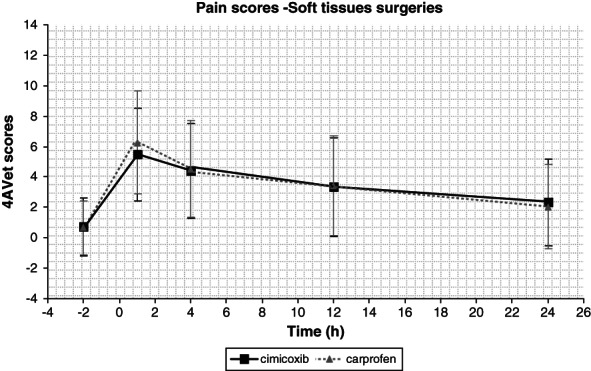
Time course of the 4A-Vet score from 2 hours before surgery until 24 hours postsurgery (soft tissues surgeries, mean and sd)

Cimicoxib demonstrated non-inferior analgesia compared to carprofen for the first 24 hours after surgery (Fig 2, rejection of inferiority P<0·0005). Cimicoxib was also shown to be non-inferior to carprofen in providing postoperative analgesia for the follow-up period of 16 to 168 hours postsurgery (excellent + good analgesia: 90·4 *versus* 82·1%; cimicoxib *versus* carprofen: rejection of inferiority P<0·005). Three dogs treated with carprofen were considered to have received inadequate analgesia (n=2 orthopaedic surgery; n=1 soft tissue surgery with two dogs requiring rescue analgesia). Inadequate analgesia was not encountered in the cimicoxib group. The owners’ observations, although not masked, confirmed that there was no significant difference in the quality of the analgesia provided during this period (Fig 3, P=0·32). Orthopaedic surgery pain scores were greater than those encountered with soft tissue surgery (Figs 4 and 5), but by 12 hours postsurgery, pain scores were lower than preoperative scores in both treatment groups (Table 5).

**Table 5:** Pain scores at inclusion

	Cimicoxib	Carprofen	Test
VAS (mm) – N=237			Kruskal-Wallis test
Mean (sd)	17·1 (24·3)	20·8 (27·0)	
Median (range)	2 (0–84)	6 (0–93)	P=0·43
4A-Vet score – N=237			Kruskal-Wallis test
Mean (sd)	2·8 (3·5)	3·3 (3·9)	
Median (range)	1 (0–13)	1 (0–14)	P=0·44
Global score of pain – N=235			Kruskal-Wallis test
Mean (sd)	3·5 (3·5)	3·3 (3·6)	
Median (range)	2 (0–14)	2 (0–20)	P=0·63

**FIG 6:**
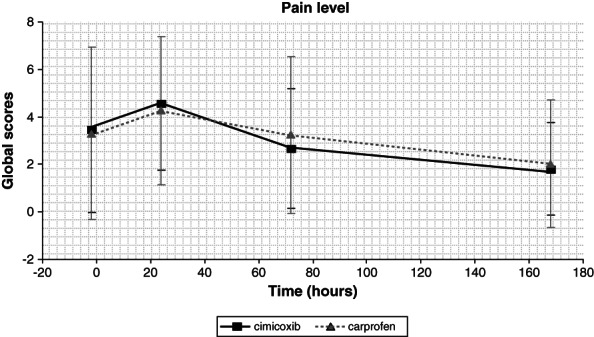
Time course of the overall pain score. No statistical difference was observed between groups (P=0·68, mean and sd)

**FIG 7:**
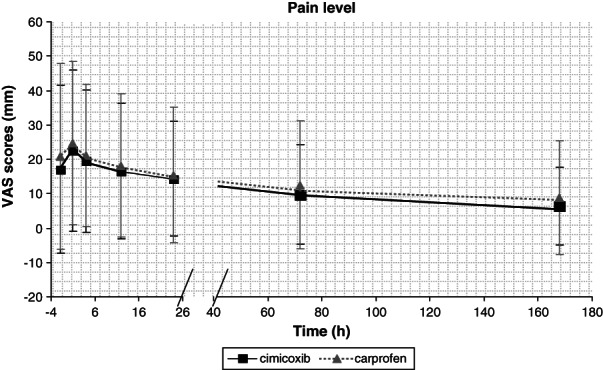
Time course of the VAS scale (mm). No statistical difference was observed between the tested groups (P=0·26, mean and sd)

In agreement with the primary efficacy criterion, there was no significant difference between cimicoxib and carprofen in controlling postoperative pain when comparing the VAS and overall pain scores (mixed model, treatment effect, P=0·26 and 0·68, respectively. Fig 6 and 7), and sedation (mixed model, treatment effect, P =0·32).

### Safety assessments

Approximately one third of the included animals in both treatment groups experienced at least one adverse event throughout the study (P=0·78, 30·7% cimicoxib *versus* 32·5% carprofen). No differences in renal, gastrointestinal, hepatic, cardiovascular or coagulation parameters between treatments groups were identified (Table 6). Two animals (one per group) experienced a serious adverse event but no causative association with the NSAID administered was identified.

**Table 6:** Distribution of gastrointestinal, hepatic, cardiovascular, renal and blood clotting disorders reported by the investigators

	Cimicoxib Dogs affected (n)	Carprofen Dogs affected (n)
Gastrointestinal disorders		
Vomiting	17	11
Diarrhoea	2	4
Obvious faecal blood	1	0
Hepatic disorders	0	0
Cardiovascular disorders	0	0
Renal disorders	1	2
Blood clotting disorders at the		
surgical site		
Haematoma	6	9
Haemorrhages	1	8

The safety of both drugs was considered acceptable based upon the sequential haematological and biochemical studies conducted. Suspicions of renal disease (n=3, urinary incontinence, polyuria/polydipsia) and blood clotting disorders (n=24, haematoma, haemorrhage) upon clinical examination were not confirmed by blood analysis. Transient postsurgical stress-associated leucocytosis was observed in both groups in conjunction with a slight decrease in the red blood cell count (RBC) 24 hours following surgery. RBC variations were considered consistent with surgical haemorrhage and administration of intra-operative fluid therapy. No significant biochemical changes were noted over the 7 day trial with the exception of a transient increase in hepatic enzymes which may have been associated with the anaesthetic agents or a result of hemodynamic changes associated with anaesthesia (Table 7).

**Table 7:** Changes in biological blood parameters from inclusion to day 7 postsurgery (mean, standard deviation)

Parameter (laboratory references)	Presurgery		24 hours postsurgery		7 days postsurgery	
	Cimicoxib	Carprofen	Cimicoxib	Carprofen	Cimicoxib	Carprofen
WBC (10^9^/L) (6·0–17·0)	11·7 (·.3)	11·4 (3·9)	17·0 (8·8)	16·9 (7·2)	11·9 (4·3)	12·6 (4·8)
RBC (10^12^/L) (5·5–8·5)	6·6 (1·0)	6·6 (1·0)	6·1 (0·9)	6·3 (1·0)	6·4 (1·0)	6·4 (0·9)
Platelet count (1×10^11^/L) (2–5)	2·8 (1·0)	2·5 (1·0)	2·6 (1·0)	2·5 (1·2)	3·2 (1·3)	3·1 (1·4)
Total neutrophil count (%) (60–77)	72·0 (9·2)	70·2 (10·0)	79·4 (7·7)	78·5 (7·9)	71·3 (9·4)	70·3 (10·6)
Urea (mmol/L) (1·68–8·3)	5·4 (2·0)	5·5 (2·1)	4·8 (2·5)	4·7 (1·8)	6·0 (2·6)	5·9 (2·1)
Creatinine (µmol/L) (35–106)	75·7 (18·3)	75·9 (21·4)	70·2 (36·2)	67·0 (18·1)	75·6 (22·4)	76·7 (22·0)
AST (U/L) (14–71)	33·2 (22·2)	31·6 (18·8)	62·3 (51·1)	59·0 (41·1)	33·0 (19·1)	28·5 (14·0)
ALT (U/L) (10–89)	52·1 (41·2)	46·4 (26·8)	56·1 (40·8)	49·8 (26·3)	51·2 (34·8)	45·3 (40·1)
ALP (U/L) (2–91)	92·0 (87·4)	96·5 (99·0)	129·7 (108·5)	138·3 (144·5)	99·8 (92·4)	101·3 (92·4)
Plasma fibrinogen assay (g/L) (2–4)	2·6 (1·3)	2·8 (1·5)	3·3 (1·5)	3·8 (3·5)	2·9 (1·2)	3·1 (2·9)
Thrombin time (s)*	16·9 (16·5)	16·9 (20·8)	18·1 (23·9)	15·0 (5·8)	14·9 (2·7)	17·0 (21·2)
Prothrombin time (s)*	9·2 (11·6)	8·5 (6·7)	11·3 (15·9)	8·5 (5·0)	7·9 (1·9)	8·3 (6·8)
Partial thromboplastin time (s)*	18·2 (22·5)	18·5 (21·8)	20·6 (27·8)	16·8 (7·6)	15·7 (5·5)	17·4 (16·1)

* Animal sample result was compared to a control sample

## DISCUSSION

As the aim of the study was to assess the efficacy of cimicoxib (Cimalgex®) under conditions found in general practice, direct or indirect pain assessment techniques not typically found in first opinion clinics (e.g. cortisol or endorphin assay, pain gauge or force plate) were not utilized. Recent veterinary research has attempted to quantify pain using a variety of techniques that are feasible in a general practice environment (Mich & Hellyer 2009). These include verbal rating scales (VRS: rating pain as none, mild, moderate or severe), numeric rating scales (NRS: assigning of numbers to a level of activity within a given category), simple descriptive scales (SDS) and VAS systems. The VAS, originally developed for human use, is a simple 100 mm line with a description of the limits of pain placed at the extremes such that 0 mm represents ‘no pain’ and 100 mm “the worst pain possible”. Studies have shown it to be more sensitive than either the VRS or NRS because it does not use defined categories, is readily reproducible and a feasible methodology for use in pain evaluation studies (Jensen *et al*. 1986). However, veterinary application of the VAS system has potential drawbacks, including reliance upon human interpretation of animal behaviour, inter-observer variability (Holton *et al*. 1998) and over-interpretation. The latter limitation can also be a feature NRS methods (Lascelles *et al*. 2001) and led to these authors dismissing both systems in favour of an SDS for a study on cat pain. In view of these shortcomings, this study utilized two alternative scoring systems in addition to a VAS system.

The first adjunctive pain assessment technique was an NRS scoring system derived from a method used in previous studies (Pibarot *et al*. 1997, Firth & Haldane 1999). This scoring system has already been tested under field conditions by the current authors (Grandemange *et al*. 2007) and enables the user to differentiate between the effects of general anaesthesia, physiological and behavioural responses resulting from pain. The second scoring systems (4AVet) has been previously validated under clinical conditions (Laboissière 2006, Holopherne-Doran *et al*. 2010) and combines a subjective pain and clinical assessment score to calculate an overall pain grade. This validated system was used for assessing the primary efficacy criterion for the first 24 hours following surgery. Benefits associated with employing multiple pain assessment systems may be limited by the possibility of discordant results although this risk could theoretically be minimized by keeping the observer consistent. Despite this potential complication, this trial provided highly consistent scores regardless of methodology as well as providing a robust comparison of the efficacy of cimicoxib and carprofen.

Randomization was stratified based upon the practice and on the type of surgical procedure thus obtaining balanced treatment groups for surgeries. This strategy also reduced variability between groups within a given practice by ensuring the investigator's assessment and surgical conditions were comparable. Variability between practices could not be avoided but this is comparable to general practice as practitioners have their own individual sensitivity towards pain management.

Carprofen was selected as the control product as it is well characterized and considered efficacious for postoperative pain (Grisneaux *et al*. 1999). Although it has been suggested that a placebo group should be included to validate the scoring system when evaluating pain (Flecknell 1994) there are significant welfare concerns associated with denying dogs postoperative pain relief under clinical conditions. Furthermore, the efficacy of carprofen *versus* placebo for the control of postoperative pain has been previously demonstrated (Grisneaux *et al*. 1999).

Although the clinician had the option to discontinue treatment after 72 hours if further analgesia was deemed unnecessary, this was only permissible in the cimicoxib group. Carprofen treatment was mandatory for 5 days postoperatively in compliance with European Marketing Authorizations and this had to be respected in a pivotal registration study.

Safety was evaluated by serial laboratory analyses of blood samples and by recording adverse events. No difference between treatment groups was observed for the incidence of adverse events and those reported throughout the study were consistent with the literature regarding NSAIDs (Lascelles *et al*. 2005, Carmichael 2011). Sporadic vomiting was the most common adverse event observed for both groups, particularly in the first 24 hours postsurgery (10 animals in the cimicoxib group *versus* 8 in the carprofen group). However, as other drugs (e.g. premedicants, anaesthetics) were also administered throughout the study, a direct link with the investigated treatments was not possible.

The results of this multi-centre field study confirm that administration of 2 mg/kg cimicoxib once a day for up to 6 days after surgery is an effective and safe method of controlling perioperative pain for dogs undergoing either orthopaedic or soft tissue surgery.
